# Routine Pediatric Vaccination in Pakistan During COVID-19: How Can Healthcare Professionals Help?

**DOI:** 10.3389/fped.2020.613433

**Published:** 2020-12-10

**Authors:** Amjad Khan, Asima Bibi, Khanzada Sheraz Khan, Ayesha Raza Butt, Hira Amin Alvi, Ada Zahra Naqvi, Saima Mushtaq, Yusra Habib Khan, Nafees Ahmad

**Affiliations:** ^1^Department of Pharmacy, Quaid-i-Azam University, Islamabad, Pakistan; ^2^Department of Healthcare Biotechnology, Atta-ur-Rahman School of Applied Biosciences, National University of Sciences and Technology, Islamabad, Pakistan; ^3^Department of Clinical Pharmacy, College of Pharmacy, Jouf University, Sakaka, Saudi Arabia; ^4^Department of Pharmacy Practice, Faculty of Pharmacy and Health Sciences, University of Balochistan, Quetta, Pakistan

**Keywords:** COVID-19, healthcare professionals, immunization, Pakistan, vaccination

## Abstract

Pakistan is still fighting to overcome vaccine-preventable diseases (VPD). The vaccination coverage in rural children remains unsatisfactory amid various barriers including price, hesitancy, and low level of awareness. COVID-19 has decreased the immunization rate in Pakistan due to restricted movements, shortage of vaccines, and low coverage. During the current pandemic, there are high risks that children may get VPD resulting in another infectious disease catastrophe. There is a dire need to put aggressive measures by the government of Pakistan in time to ensure the optimal vaccine coverage. Public education programs for immunization, telehealth services, the involvement of community pharmacies, and the drive-through vaccination system may help to enhance the vaccination rate during the ongoing health crisis.

## Background

Pakistan is at a crucial stage in the immunization world. The birthrate is 7.8 million per year; more than one third are not getting vaccination on the first day and are at risk of developing vaccine-preventable diseases (VPD). There are many vaccine-preventable childhood diseases including polio, influenza, mumps, measles, tuberculosis, tetanus, diphtheria, and pertussis. The expanded program on immunization (EPI) in Pakistan, was developed in pursuance of the 18th constitutional amendment, is aimed to vaccinate children and pregnant women to prevent these deadly diseases. During COVID-19, the vaccination rate is decreased, and it can result in children's exposure to vaccines preventable deadly diseases. Furthermore, global lockdown leads to a shortage of pentavalent vaccines and medical supplies e.g., syringes, needles, cold chain commodities (1).

The global COVID-19 pandemic has fetched unparalleled changes in the health care system and has shown damage to human lives. The nationwide lockdown reduced the risk of transmission of COVID, but it has devastated routine maternal and child health services including immunization. Different vaccination drives have been postponed due to increased risk of disease outbreaks, resulted in un-vaccination or under-vaccination, putting their lives at risk of VPD (2). According to the WHO “Vaccines are one of the most powerful tools in the history of public health, and more children are now being immunized than ever before” but the pandemic has put those gains at risk (3). The preliminary data for the first 4 months of 2020 showing a substantial drop in the number of children completing three doses of the vaccine against diphtheria, tetanus and pertussis (DTP3), this reduction in DTP3 coverage happened for the first time in 28 years & it is noted that due to COVID-19 pandemic almost 30 measles vaccination campaigns were at high risk which could result in further outbreaks in 2020 and later on (3). According to the center for disease control and prevention (CDC); 419 million illnesses, 8 million hospitalizations, and 936,000 deaths were prevented by vaccination among children born between 1994 and 2018 (4). In 2018, Measles infected an estimated 10 million and killed 140,000 (5) Several statistical models have demonstrated that 28,219,100 measles cases and 535,600 deaths occurred in 2000 and 7,585,900 cases and 124,000 deaths reported in 2017 (6). Various health organizations estimated that over 117 million children in 37 countries may miss out on receiving life-saving measles vaccine and its campaigns already been delayed in 24 countries and more will be postponed as COVID-19 surges (7). Vaccination campaigns in Pakistan have experienced various hurdles including religious misbelieves, vaccine hesitancy and acceptability, terrorism and poor coverage. It is estimated that 147 cases of polio were reported in 2019 and so far, this year, authorities have recorded 41 cases. It is pertinent to mention that around 40 million Pakistani children have missed the polio vaccination program in April due to the coronavirus outbreak (8).

This outbreak reminds us the importance of routine immunization for children, if there is a vaccine available for a VPD, then children must adhere to the vaccination schedule. Currently, the healthcare system in Pakistan is overwhelmed and authorities are doing their best to contain the virus (9). However, vaccination programs might be neglected during the ongoing pandemic. Proper assessment is required to engulf potential harms due to the weak immunization system in Pakistan raised by COVID-19.

## Strategies for Vaccination During COVID-19 in Pakistan

Globally, initiative measures have been taken to control the transmission and to reduce the influence of the COVID-19 on health-care systems. For maintaining the scheduled childhood immunization during this pandemic, concerned authorities and healthcare providers may adopt some novel approaches. Healthcare providers including physicians, pharmacists, nurses, and lady health workers/visitors can play a vital role in providing routine immunization and to educate the hesitant parents for taking precautions while bringing their children for immunization. In this context, we recommend the following strategies which can be proved effective during the current situation.

## Role of Health Authorities

As vaccination centers are closed at various healthcare facilities throughout the country to minimize the viral spread which is the major cause of missing pediatric immunization. To overcome this problem, the concerned health authorities should implement the adoption of drive-through vaccine services at vaccination centers. This strategy can avoid huge gatherings and support social distancing. At the same time, the authorities make sure the availability of vaccines inventory in hospitals and community pharmacies across the country. Recently, the role of community pharmacists as vaccinator has been proved to be promising. Since pharmacies provide services to the limited local area, utilization of community pharmacists for vaccination campaigns aid to achieve optimum coverage.

## Establishment of Telehealth Services

Telehealth services have provided an enabling tool to maintain patient continuity and management of patients during COVID-19. These services could be of great help in providing accurate information to the parents on routine pediatric vaccination and the available facilities at different vaccination centers in the current pandemic. Telehealth services should be established in different government and private healthcare facilities. All related information including contact details of these telehealth centers should spread throughout the country. Advertisements on news channels are the most effective source of awareness for the public. Appointment procedures and step by step guidelines should be displayed on hospitals/centers websites and can be conveyed to the public through news channels and social media forums. The public gets benefits from these services at affordable prices. After physician-parent telephonic or video-teleconferencing communication, healthcare teams can actively participate in ensuring door to door immunization and to reach those areas where children missed their scheduled vaccination.

## Registration and Prioritizing Children With Missed Routine Doses

The government should engage healthcare professionals in the identification and registration process of children with missed routine doses. Priority should be given to these children in the current pandemic situation.

## Immunization Campaigns

Conduction of mass vaccination campaigns at an affordable cost can cover a large population even in areas where other facilities are strenuous and challenging. In a short time, the immunization campaigns can give better results.

## Drive-Through Vaccine Services

Healthcare professionals should recommend parents to adopt drive-through vaccine services for their children where appointments can be provided on call and they may reach on location at the scheduled time. Walk-through sanitization gates should install at immunization centers. Precautions are a must to follow for securing parents, children, and staff by utilizing masks and gloves, screening patients before they come in, taking temperatures on location, and utilizing separate waiting areas to prevent overcrowdings for immunization visits.

## Public Awareness

Public awareness on the importance of vaccination plays an important role to increase the coverage. Health authorities in Pakistan should take necessary measures to arrange awareness campaigns at a mass level. Since Pakistan is lifting lockdown in a stepwise manner, awareness campaigns can be targeted in offices, markets, shopping malls, and hypermarkets. Public health educators should ensure that everyone gets information on the importance of vaccines and the harms of missing the recommended dosages.

## Data Availability Statement

The original contributions presented in the study are included in the article/supplementary materials, further inquiries can be directed to the corresponding author/s.

## Ethics Statement

Ethical review and approval was not required for the study on human participants in accordance with the local legislation and institutional requirements. Written informed consent for participation was not required for this study in accordance with the national legislation and the institutional requirements.

## Author Contributions

AK, AB, KS, AR, HA, AZ, SM, YK, and NA made substantial contributions to the conception and design of this study. KS, AR, HA, and AZ performed the literature search. AK, AB, and SM drafted the manuscript. AK, YK, and NA were involved in critical revision for important intellectual content. The study is supervised by AK. All authors read and approved the final manuscript.

## Conflict of Interest

The authors declare that the research was conducted in the absence of any commercial or financial relationships that could be construed as a potential conflict of interest.
